# Intake of Novel Red Clover Supplementation for 12 Weeks Improves Bone Status in Healthy Menopausal Women

**DOI:** 10.1155/2015/689138

**Published:** 2015-07-21

**Authors:** Anne Cathrine Thorup, Max Norman Lambert, Henriette Strøm Kahr, Mette Bjerre, Per Bendix Jeppesen

**Affiliations:** ^1^Department of Endocrinology and Internal Medicine, Aarhus University Hospital, Tage-Hansens Gade 2, 8000 Aarhus C, Denmark; ^2^Department of Obstetrics and Gynecology, Vendsyssel Hospital, Bispensgade 37, 9800 Hjørring, Denmark; ^3^Department of Clinical Medicine, Aalborg University, Søndre Skovvej 15, 9000 Aalborg, Denmark; ^4^The Medical Research Laboratory, Department of Clinical Medicine, Faculty of Health, Aarhus University, Nørrebrogade 44, 8000 Aarhus C, Denmark

## Abstract

*Objective*. To investigate the effect by which daily consumption of a novel red clover (RC) extract influences bone health, inflammatory status, and cardiovascular health in healthy menopausal women. *Design*. A 12-week randomized, double-blinded, placebo-controlled trial involving 60 menopausal women receiving a daily dose of 150 mL RC extract containing 37.1 mg isoflavones (33.8 mg as aglycones) or placebo. *Methods*. Bone parameters were changes in bone mineral density (BMD), bone mineral content (BMC), and *T*-score at the lumbar spine and femoral neck. Bone turnover (CTx) and inflammatory markers were measured in plasma and finally blood pressure (BP) was evaluated. *Results*. RC extract had positive effect on bone health, and only the women receiving the placebo experienced a decline in BMD (*p* < 0.01) at the lumbar spine. *T*-score at the lumbar spine only decreased in the placebo group (*p* < 0.01). CTx decreased in the RC group with −9.94 (±4.93)%, although not significant. *Conclusion*. Daily consumption of RC extract over a 12-week period was found to have a beneficial effect on bone health in menopausal women based on BMD and *T*-score at the lumbar spine and plasma CTx levels. No changes in BP or inflammation markers were found and no side effects were observed.

## 1. Introduction

Menopause is a natural occurring part of life characterized by amenorrhea due to the cessation of the ovarian function causing reduced circulating estrogens levels. During and after menopause, an intense bone loss occurs which over time can lead to osteopenia. Osteopenia is often asymptomatic, generally presenting no or mild clinical symptoms until development of fractures and osteoporosis. Osteoporosis is a metabolic disorder, characterized by low bone mass and density, causing bone fragility and increased risk of bone fracture. The maintenance of bone homeostasis in mammals is influenced by estrogens and the decline in endogenous estrogen (especially 17*β*-estradiol) during and after menopause. It results in enhanced bone resorption accompanied by impaired bone formation, regeneration, and increase of fat tissue in the bone marrow [1, 2]. The treatment of choice to remedy and prevent osteoporosis and consequently occurrence of fractures was previously hormone replacement therapy (HRT) [3, 4]. However, in 2002 the first publication [5] from the Women's Health Initiative trial yielded statistically significant increases in cases of breast cancer and cardiovascular events in HRT-treated women. This prompted sudden changes to recommendations concerning HRT. Due to the critical risk-benefit profile, pharmacological treatment cannot be used in cases of low osteoporosis risk, and osteopenia itself is not a criterion for pharmacotherapy under current international guidelines [6].

A range of antiresorptive or anabolic options are available for the treatment of postmenopausal osteoporosis reducing the fracture risk [7]; however, the existing treatment program for osteoporosis is associated with a significant risk of adverse effects [8, 9]. It is of both medical and scientific interest to find alternative therapies that have a milder action which provide the same beneficial results and less adverse side effects.

Within the last decade the use of natural supplements has become more widespread in the search for viable alternatives to existing treatments [10–15]. A compelling alternative is phytoestrogens which have shown promising results as an alternative medicine. Phytoestrogens are secondary metabolites that can induce biological responses in vertebrates and can mimic or modulate the actions of endogenous oestrogens, usually by binding to the estrogen receptors (ERs) [16]. ERs are steroid receptors but are unique in their ability to interact with a variety of compounds [17]. Phytoestrogens are believed to work by binding to ERs (two subtypes exist, ER*α* and ER*β*) on cell membranes, similar to the body's own steroid oestrogens [18]. The ER*β* is widely expressed in the body and the predominantly receptor in trabecular bone [19], but in the breast and uterus tissue the oestrogenic effects are mediated by ER*α*. The distribution of the ERs opens up the possibility to target specific tissues and thereby avoid certain negative effects of the classical estrogen pathway [20]. As selective oestrogen receptor modulators (SERMs) compounds, phytoestrogens may be considered as a therapeutic option in the prevention of osteoporosis [21], because these plant hormones can mimic the endogenous estrogens but lack the adverse side effects of HRT [5, 6].

Isoflavones make up the most common and well-known form of phytoestrogens, and in particular isoflavones (genistein and daidzein) from soy have received considerable attention in the management of postmenopausal bone loss [10, 22, 23]. Although the relative affinity of all isoflavones to the ER is lower than for estradiol, they can induce conformational changes that result in transcription activation. When absorbed into the body, phytoestrogens are able to behave similarly to weak natural estrogen; however ER binding can result in both agonist and antagonist actions. In the bone tissue an agonist activity is preferable as low levels of natural oestrogen will lead to weakened activity.* In vitro* and animal studies have revealed that phytoestrogens can enhance bone formation and increase bone mineral density and levels of alkaline phosphatase, osteocalcin, osteopontin, and *α*1(I) collagen. Furthermore, phytoestrogens are shown to suppress the rate of bone resorption and enhance bone formation rate [13, 24]. A large array of variables is known to influence bioavailability, metabolism, and ultimately physiological effects of phytoestrogens.* In vitro* and animal studies have suggested that phytoestrogens can have a significant effect on oestrogen-sensitive tissues, but the results from human clinical trials remain inconsistent [10].

Red clover (RC) contains phytoestrogens (i.e., isoflavones: formononetin, biochanin A, genistein, and daidzein), which bind to the estrogen receptors and can elicit a weak agonist, antagonist, or partial agonist antagonist response. The effect depends on the compound and the target tissue. The isoflavones are activated when the glucose residue is removed by the bacterial enzymes in the gastrointestinal flora. Thus, the bioavailability of phytoestrogens is particularly dependent on the bacterial flora, which varies considerably between individuals [10]. Unlike soy, RC has a high content of both formononetin and biochanin A, and studies in ovariectomised rat models have shown that formononetin contributes to bone formation process by stimulation differentiation of osteoblasts [14, 15, 25]. In addition formononetin has a higher estrogenic activity than daidzein [26]. Biochanin A has been demonstrated to induce a reduction in resorption of bone at the femoral neck when given to ovariectomised rats [27].

The study is, to our knowledge, the first to investigate the effect of daily consumption of a novel RC extract (rich in isoflavones due to the new fermentation process) which can help maintain bone mineral density, as determined by bone mineral density via DEXA scans, inflammatory status, and cardiovascular health in healthy menopausal women.

## 2. Subjects and Methods

### 2.1. Study Design

The study was conducted according to the guidelines in the Declaration of Helsinki and approved by the Danish Ethical Committee (M-20110267) and the Danish Data Protection Agency. According to the International Committee of Medical Journal Editors, the study protocol was registered at ClinicalTrials.gov (NCT02028702). The study was carried out as a 3-month, randomized, double-blinded, parallel designed trial. The primary endpoint was change in frequency and intensity of hot flashes, sleep disturbances, and flush related sweat (to be published elsewhere). Secondary endpoints were change in BMD as assessed by DXA, change in the levels of C-terminal telopeptide of type 1 collagen (CTx), blood pressure, and inflammatory markers.

**Figure 1:**
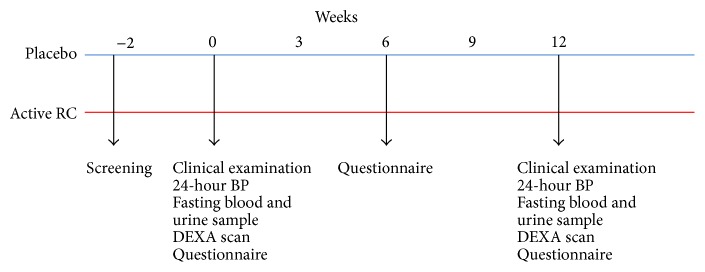
The design of the intervention study. The far left shows the 2 groups, in the top the placebo group (blue) and those receiving RC extract (red) below. Screening took place 2 weeks prior to project start. At weeks 0 and 12 fasting blood and urine samples were taken, 24-hour blood pressure (BP) was measured, and DEXA scans were performed. At weeks 0, 6, and 12 participants filled out questionnaires. Randomization took place at week 0.

The study involved two arms (Figure 1):

1. Red group: active treatment (RC extract),
2. Blue group: placebo.

The RC extract and placebo were handed out once every two weeks followed by a verbal meeting to ensure the participants compliance during the project. Participants in each group received a daily dose of 150 mL (75 mL in the morning and 75 mL in the evening) extract or equivalent dose of placebo equating to an approximate total of 13.5 liters over 90 days. During each extract collection day, each participant received either RC extract or placebo as required, approximately 2–4 litres per visit as necessary (accounting for losses). The participants would return empty 2-litre containers, which were collected by the research team as a measure for compliance. RC extract and placebo were packed in identical sealed brown cardboard cartons and marked with a red or blue cross, corresponding to either the red or blue group. The participants would incorporate the study extract into their normal habitual morning and evening diet. Both participants and research team were blinded to the content of the cartons throughout the study process. Participants were randomized, by computer generation, at the initiation of the study, but were not informed about their randomization allocation; however they were all required to consume 150 mL extract or equivalent placebo daily. The research team was also blinded and the active or the placebo group was revealed after the study period to both participants and researchers.

### 2.2. Participant Recruitment and Screening

Participants were recruited from the general population in the Northern Denmark Region via press release and by advertisement within the local paper. Interested women were screened for eligibility by telephone at first, and if they met the inclusion criteria, they were invited to the clinic for a screening visit. The screening visit consisted of an extended interview followed by a blood sample. Basic anthropometric measurements were also recorded. The inclusion criteria were as follows: women with acknowledged problems with menopausal symptoms (>5 hot flushes per day); FSH level ≥35 UI/L; 40–65 years of age; BMI 20–40 kg/m^2^; and having a reported variable cycle length of >7 days different from normal. The exclusion criteria were as follows: participation in other clinical trials within the last 6 months; cardiovascular, chronic liver, thyroid, or kidney diseases; a history of cancer; a disease or condition, which may influence the participants ability to follow the study protocol; alcohol or drug abuse; use of hormonal contraceptives within the last 3 months; reported excessive dietary intake of isoflavone-rich foods; and finally blood pressure above >160/110 mmHg.

Participants provided written informed consent prior to enrolment into the trial and eligibility or exclusion was assessed by the research team based on the screening visit and the following results.

**Figure 2:**
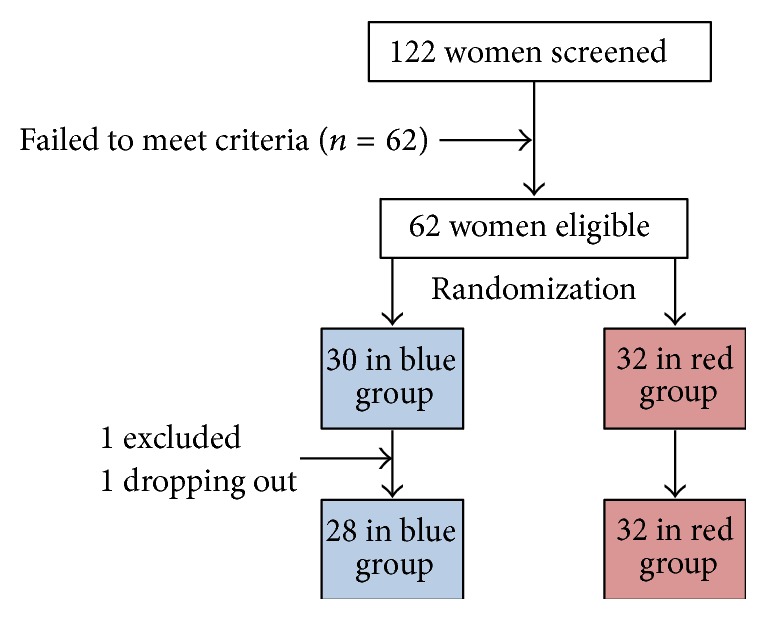
Flow of participants through the study.

122 women underwent the screening visit, but only 62 women met the inclusion criteria and were enrolled in the study. One participant dropped out due to personal reasons, and another participant was excluded during the medical exam due to undisclosed information regarding her physiological status. A total of 60 women successfully completed the trial (Figure 2).

The participants were instructed to keep physical activity, smoking, and drinking habits constant and were told to maintain their normal diet and finally to abstain from any change in intake of nutritional supplements during the study period.

### 2.3. Study Assessments

The study included measurements of body composition, BP, fasting plasma samples, and DEXA scans. It was carried out at Center for Clinical Research, Vendsyssel Hospital, Aalborg University. Study researchers met the participants once every 2 weeks for extract dispensing and to perform motivational interviews, monitor compliance, and discuss any individual difficulties that might have occurred. Before the intervention and at weeks 6 and 12 a thorough questionnaire on dietary intake, exercise habit, alcohol and tobacco use, and lifestyle choices was used to ensure no alterations in the participant's lifestyles during the intervention period.

*Body Composition*. Before the intervention and at week 12 standing height, weight (participants lightly clothed), and waist circumferences were measured.

*Blood Pressure*. 24-hour blood pressure measurements were performed before the intervention and at week 12 using a Spacelab monitor (Spacelabs Medical, Redmond, WA). The participants would come into the clinic in the morning and a trained study researcher would apply the blood pressure monitor to the right upper arm. The monitor was preprogrammed to measure the blood pressure hourly [28], and the participant was given a thorough oral instruction not to move or talk during the measurements and relax their arms in an extended position during measurements. The participants were also given a form to write down any unusual activity during the 24 hours of measurements as well as their individual bedtimes. Participants were advised that they could carry out their usual activities but avoid strenuous exercise. Daytime was defined as measurements from 6:00 h–23:00 h, and night-time was within the timeframe of 23:00 h to 6:00 h.

Fasting plasma samples were collected at weeks 0 and 12. The participants would come into the clinic after an overnight fast and submit a blood sample after which they were offered breakfast.

Measurements of BMD in all participants were assessed by DEXA at baseline (week 0) and after the 12-week study period (week 12). All scans were performed by a trained radiographer using the XR-800 DEXA scanner (Norland Cooper, Surgical, USA, illuminates software version 4.2.4). The participants would come into the Orthopedic Department at the hospital and each scan lasted for 20 min. During this time, measurements of both bone mineral content in grams (BMC) and bone mineral density (g/cm^2^) at the lumbar spine region (L2–L4) and at the femoral neck were assessed. The coefficient of variation of repositioning was 1% at the lumbar spine and 2% at the hip.

### 2.4. Biochemical Analysis

A fasting blood sample was collected at weeks 0, 6, and 12 using an evacuated-tube system and transferred to EDTA or lithium heparin evacuated tubes, temporarily stored on ice, and centrifuged at 4°C at 3500 g within 30 min. Plasma was then separated into aliquots and stored at −80°C. All samples from each subject were analyzed within one batch to reduce interbatch variation after study completion. All laboratory analyses were blinded, and all analytic procedures were carried out in accordance with the manufacturer's instructions.

As a marker of bone resorption we analyzed C-terminal telopeptide of type 1 collagen (CTx) in plasma before and after the study period. The analysis is a standard laboratory method (sandwich immunometric assay, CV 5%) at the Department of Clinical Biochemistry (Aarhus University Hospital, Aarhus, Denmark).

Plasma cytokines (IL-1b, IL-2, IL-4, IL-5, IL-6, IL-10, IL-12, IL-13, and IFN-*γ*) were measured using the Precision Pro Human Cytokine Assay (Bio-Rad, Hercules, CA, USA), according to the manufacturer's instructions. Detection limits were below 1 pg/mL with an intra- and interassay CV below 10% and 15%, respectively. The responses were analyzed using the BioPlex Manager 6.0 software (Bio-Rad).

### 2.5. Red Clover Extract

The RC extract was produced and provided by Herrens Mark v/Michael Mohr Jensen, Nørre. Aaby, Denmark. Red clover crops were harvested approximately 6–8 months before being used in the study. Post-harvest processing requires the plant material to be transported to rust-free drums. Following this step, the RC mass is pressed and the resulting extract is pumped into large tanks where it is stored at 3–5°C. Aqueous extracts are left within the tanks, and lactic acid bacteria are added to the RC extract to induce fermentation for 6 months. During storage, the fermenting extract is routinely moved to a new tank every 6–8 weeks, allowing the pH to gradually fall, and the end pH of the extract lies at 4, which helps preserve the extract. The novel patented fermentation process (PA 2013/00233DK) is integral for the hydrolysis of isoflavone glycoside and glycoside compounds, which improve the bioavailability of the plant derived phytoestrogens.

In order to improve the likability and acceptability of the RC extract, a natural calorie-free sweetener stevia and a natural sugar-free raspberry/orange flavour were added. Specifically, 90 litres of either water (placebo) or RC extract was sweetened with 18 g of stevia and 6.3 litres of raspberry/orange flavouring to improve the taste. Due to the brown colour of the RC extract, brown food colouring was also added to the water-based placebo to achieve likeness in appearance to RC extract. The placebo was a liquid indistinguishable from RC extract also containing stevia and sugar-free raspberry/orange flavour, which enabled blinding of the study for both participants and the research team.

The postfermentation isoflavone composition and quantification analysis of the final extract was performed by DB Lab (DB Lab A/S, Odense SØ, Denmark). Quantification of compounds was assessed using high performance liquid chromatography with ultraviolet and mass spectrometry detection in a Summit LC/MS system (Dionex Corporation, Idstein, Germany). Standards for four of the primary isoflavones (genistein, daidzein, formononetin, and biochanin A) and the latter two glycoside derivatives (ononin and sissotrin) were obtained from Sigma Aldrich Denmark A/S (Brøndby, Denmark).

Based on the analysis (Table 1) of the RC extract women in the active RC group were given a dose of 37.1 mg isoflavones per day, of which 33.8 mg was aglycones.

### 2.6. Statistical Analysis

Statistical analysis included all participants who completed the study and from which appropriate material was obtained. Data was analyzed using StataIC statistical software (version 11.2; StataCorp LP, College Station, Texas, USA), and graphs were created by GraphPad Prism version 4 (GraphPad Software Inc., San Diego, CA, USA). Normal distribution of the data was checked by QQ-plots and histograms for the two groups separately. Baseline comparisons were assessed by unpaired Student's *t*-test, and absolute within-group changes from baseline were calculated after the 12-week study period and tested by paired *t*-test. Changes are presented as mean ± SEM. In all cases, *p* ≤ 0.05 was considered significant. Power calculations were made on primary menopausal related symptoms (frequency and intensity of hot flushes). The minimum number of participants was determined by power calculations to be 62 (31 in each group), assuming a reduction in percentage of women experiencing hot flushes by at least 15% (minimal clinically important difference determined by Lipovac et al. [29]). An expected drop-out rate is 15%, power is 80%, SD is 3.3, and level of significance is 5%.

## 3. Results

### 3.1. Baseline Characteristics

A total of 60 women completed the study, and after randomization, subjects in the two groups were comparable with respect to all measured baseline characteristics (Table 2). Average age was 52.5 (±0.5) years and average BMI was 25.7 (±0.57) kg/m^2^ at baseline.

### 3.2. Extract Isoflavone Content

The results of the postfermentation isoflavone composition and quantification analysis revealed that the majority of the isoflavones were as aglycones, although total conversion was not achieved due to the presence of ononin and sissotrin glucosides (Table 1).

### 3.3. Bone Mass and Density

**Figure 3:**
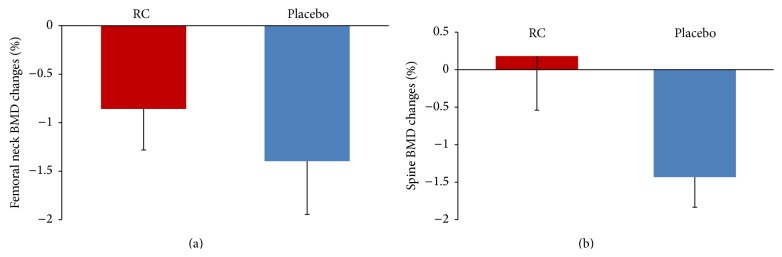
Percentage change (Δ) in femoral neck (a) and lumbar spine (b) BMD after 12-week intervention with RC and placebo. Data are presented as mean (±SEM). Femoral neck: RC group (*p* < 0.05) and placebo (*p* < 0.05). Lumbar spine: RC group (NS) and placebo (*p* < 0.01). All statistical calculations are within-group difference from start to end. RC = red clover.

We found a significant intragroup decrease in femoral neck BMD after 12 weeks of intervention in both the RC group (−0.86 ± 0.42%, *p* < 0.05) and the placebo group (−1.40 ± 0.55%, *p* < 0.05). In the lumbar spine region only the placebo group experienced a significant decline in BMD of −1.4 (±0.40%, *p* < 0.01) whereas the RC group had a percentagewise increase of 0.18 (±0.72%, *p* > 0.05) after the 12 weeks (Figure 3).

In addition, a small decline in femoral neck BMC was apparent in both groups (Table 4). In the RC group mean decrease in femoral neck BMC was −1.07 (±0.47%, *p* < 0.05), where the mean decrease in femoral neck BMC in the placebo group was −0.85 (±0.51%, *p* > 0.05). As expressed by *p* values only the result from the RC group was statistically significant. Regarding lumbar spine BMC, no significant changes were observed (Table 4).

The *T*-score is a comparison of the participants BMD results compared to a healthy 30-year-old adult; so a *T*-score of 0 means that the BMD is equal to the norm for a healthy young adult. The femoral neck *T*-score changed significantly within both the RC and placebo groups, with a mean percentage change in the RC group of 3.98 (±24.12%, *p* < 0.05) and the mean percentage change in the placebo group of −57.77 (±32.89%, *p* < 0.05). Regarding the *T*-score for the lumbar spine we saw a mean percentage change of −1.71 (±10.38%, *p* > 0.05) and −18.41 (±8.54%, *p* < 0.01) in the RC and placebo, respectively.

### 3.4. Biochemical Analysis

The bone resorption marker CTx increased by 1.17% (±5.95, *p* > 0.05) in the placebo group and decreased by −9.94 (±4.93, *p* > 0.05)% in the active RC group, but the within-group differences were not statistically significant.

### 3.5. Blood Pressure

The results from the 24-hour blood pressure (Table 3) reveal no significant changes in systolic or diastolic blood pressure.

### 3.6. Inflammation

No significant differences were found within groups regarding the analysed cytokines in the RC and placebo group (Table 5). No significant differences were observed between baseline values (week 0) in the two groups.

### 3.7. Compliance

Compliance measured as counting of the returned used empty 2-litre containers (8 in total per participant) showed a 100% compliance in both groups.

## 4. Discussion

In this randomized, placebo-controlled intervention study, which included 60 healthy menopausal women, we found that a daily supplement of red clover extract containing isoflavones, mainly in the aglycone form, had positive effect on bone health. The spinal BMD, *T*-score, and CTx levels all improved in the group receiving the active RC supplement, but no effects were found on inflammatory markers and blood pressure. The isoflavones used in our study were derived from red clover and found in the extract product after it had been processed by a novel patented method.

Decline in bone mass will naturally occur during menopause due to decrease in estrogen production. Women reporting vasomotor symptoms are associated with having an even lower BMD than those who do not experience symptoms [30, 31]. As the first and most prominent stage of accelerated bone resorption occurs during menopause, women during this period may provide a better test group for therapies targeting bone mineral resorption as the higher BMD resorption may make them more sensitive to beneficial treatments. The average *T*-score at baseline in both groups was for both femoral neck and lumbar spine below 0, meaning that the BMD in the bones were lower than in a healthy 30-year-old woman. Osteopenia is diagnosed when the *T*-score is between −1 and −2.5 [32], so looking at the participants as a combined group, their mean *T*-scores reveal that they did not have osteopenia. With the average age of the participants being in their early fifties, it was predictable that their *T*-score was negative. We saw a large percentage decline in the femoral neck *T*-score in the placebo group, meaning that their bone strength declined over the intervention period. The opposite was seen in the RC treated group, where the femoral neck *T*-score increased over the 12 weeks.

DEXA scan was performed in both lumbar spine (L2–L4) and femoral neck area, but the greatest effect on bone status was seen in the lumbar spine. The decline in estrogen production during menopause might first present low BMD in lumbar spine before the femoral neck supplementation with estrogen or even phytoestrogens may benefit the lumbar spine in the short term to a greater degree when compared to the femoral neck. The lumber spine contains higher trabecular tissue in contrast to the femoral neck and could retain a shorter response time to treatment than the femoral neck. A treatment window of three months may only have enabled attenuation to occur in certain locations with faster remodelling rates; for example, spinal remodelling is shown to occur at a higher rate than at the hip, as is apparent in the present study [33, 34]. In the lumbar spine the anticipated natural decline in BMD over time was observed in the placebo group and results showed a significant decline in BMD. Interestingly, the mean lumbar spine BMD change in the treatment group from baseline to week 12 of intervention was slightly positive, which demonstrated that treatment with red clover extract had an attenuating effect on bone resorption. Perhaps giving a longer term treatment might even stimulate bone formation and/or have had a greater effect on the hip.

One peculiar finding within the study was that spinal BMC in the placebo group did not significantly decrease with spinal BMD. We observed a nonsignificant increase of spinal BMC in the placebo group; however similar result has also been observed in other studies, supporting the theory that BMC is not directly proportional to bone area. As a consequence, changes in bone area and BMC can occur independently of one another; however, the interpretation and relationship of BMC and BMD at skeletal sites needs to be further investigated. It is worth considering that given a constant BMC, any increase to areal bone size will decrease the BMD [35, 36]. In our study, data suggests that the placebo group developed more porous bone structures, which have the same BMC but are less densely assembled together as a consequence of resorption phase bias. Moreover, other studies have shown that the effect of soy isoflavones on BMC is greater in the longer duration since menopause, indicating that a more stringent control of our participants in selecting for menopausal stage may yield better results when assessing BMC [37].

Despite the large numbers of studies of isoflavones derived from soy and partly red clover, due to the heterogeneity of the human trials, differences in both the types of isoflavones used and the form in which they were provided (glycoside compared to aglycone), differences in isoflavone metabolism, and so forth, it is still not clear which specific products and what dose will provide the best health benefits regarding bone metabolism effects [38–43]. A number of studies have attempted to address whether isoflavones can prevent and/or treat menopausal and postmenopausal bone loss. Most of the studies conclude that current evidence is not sufficient to make recommendations regarding the effects of isoflavones on bone health [10, 44–46].

In plants, isoflavones are inactive when present in the bound form as glycosides, but when the sugar residue is removed these compounds become activated and only the aglycones can be absorbed [47]. During the preparations of the RC extract used in this study, adding of lactic acid bacteria and the processing for 6 months give a high level of isoflavones in aglycone form contributing to a higher bioavailability and hence a higher physiological effect. However, the bioavailability of phytoestrogens also depends largely on the intestinal flora because bacterial enzymes in the intestine transform isoflavones into different metabolites. Approximately one-third of humans microbial gut flora also naturally transform daidzein (aglycone form of daidzin) into equol, which is adsorbed in the intestine and is 10–100-fold more estrogenic than both daidzein and genistein [48]. Equol-producers were not categorized in our study but in another clinical trial investigating the effect of soy isoflavones on menopausal women and their analyses on a subgroup of equol producing women showed no benefit when compared to nonequol producing menopausal women [49].

Participants did not receive the entire dose of daily RC all at once but one dose in the morning and one in the evening. This might give a higher uptake in the gastrointestinal tract as the body has an upper limit regarding the absorption rate of isoflavones and their derived metabolites. If the dose was further spread out during the day, there might be an even higher uptake, but this might be too inconvenient to the participants and could result in lower compliance. We also have no knowledge as to which isoflavones or derived metabolites precisely induce positive health effect on the bones or the extent of synergistic effects. However, RC has a high content of formononetin (Table 1) and studies have shown that formononetin has positive effects on bone health.

It is recommended that the duration of the interventions using bone formation markers as an indicator of bone turnover last for at least 6 months [50]. Due to the relative short timeframe of our study, we choose only to look at CTx, a bone resorption marker. We found a nonsignificant trend for decreased plasma CTx, in the group receiving the active red clover extract, suggesting that bone resorption is decreased. The placebo group experienced no changes in CTx levels, which indicates that they experience no changes with respect bone resorption.

No changes were found in regard to 24-hour blood pressure in either RC or placebo group, but as seen in Table 2 the blood pressure of the participants was within the normal range with no or very little room for improvement. It might have been better to measure changes in total vascular resistance and arterial stiffness as opposed to blood pressure in participants with an already healthy cardiovascular profile [51].

Oestrogen also has an effect on the synthesis and activity of various inflammatory cytokines, and a lack of oestrogen therefore leads to increased levels of inflammatory cytokines [52, 53]. Changes in level of low-grade inflammation could also have an effect on bone metabolism; however, no effect on inflammation markers could be demonstrated after 12 weeks of intervention with RC extract. The positive effect of red clover on bone health in this study could not be explained by anti-inflammatory effects. Interestingly, this suggests that a component of isoflavone action on bone may be independent of inflammation markers.

Regarding the safety of the active group with a daily intake of 37.1 mg isoflavones, recently a 2-year trial accessing the safety issues related to a high isoflavone intake (80–120 mg soy hypocotyls isoflavones) concluded that daily supplementation for 2 years is associated with minimal risk in healthy menopausal women [54]. No severe events were observed in our study during the 12 weeks of intervention.

Age, race, and dietary components like carbohydrate and fat content in the diet, type of dietary fat, presence of probiotics, and so forth all influence the isoflavone metabolism [55]. The participants were instructed not to change anything regarding lifestyle and dietary habits in the intervention period, and questionnaires confirm that the eating pattern is similar before and during the study period. None of the participants had high intake of isoflavone containing foods as this was part of the inclusion criteria.

Body weight plays an important role in the rate of menopausal bone loss, because rates of bone loss are 35% to 55% slower in women with higher BMI. Increased production of estrogens by adipose tissue may contribute to the association between BMD and body weight. Our participants had a mean BMI revealing that they were normal weight to slightly overweight. However, participants were not overweight enough for it to have any influence on the BMD [30].

There is a wide disparity in BMD, bone turnover, and fracture incidence across ethnic groups [56, 57], but the women in our study were all Caucasian from the northern part of Denmark, so this was not a factor we had to take into consideration.

The strengths of the study include the design, a randomized, placebo-controlled, double-blinded study with a good adherence rate. The participants had a great interest in the study due to personal discomfort caused by their menopausal status, which contributed to the low drop-out rate and 100% compliance according to the returned boxes. The RC extract and placebo intake were very convenient for the participants, and they were able continue with their normal life during the intervention period. The method used for diagnosing osteoporosis is dual-energy X-ray absorptiometry (DEXA) scan, which can determine bone density at whole body level or at specific areas such as the hip and lower spine, the typical and most vulnerable areas of the body. The same diagnostic methods as used in the clinic and doctors standard practice [8] were utilised to characterize and diagnose osteoporosis which were used in this study.

The short intervention period prevents us from drawing any conclusions about the long-term effect of red clover on bone metabolism, but this would be interesting to look into in the future.

RC extract intake is convenient and can easily be incorporated into everyday life. In therapeutic use, RC extract might be categorized as a preventive treatment or a prophylactic preparation regarding bone status and menopausal symptoms.

Our study showed that healthy nonosteopenic women had a beneficial effect on bone status when consuming the RC extract. Since the treatment of osteoporosis and osteoporotic fractures has a huge impact on individual recovery and the national health budget [8], it is important to have a greater focus on prevention of bone loss in relation to postfactum treatment.

Further studies of longer durations comprising populations at risk of osteoporosis are needed to confirm these positive effects of red clover extract, but a long-term study is currently being conducted.

To conclude, intervention of daily consumption of RC extract with a high content of bioavailable aglycone isoflavones over a three-month period was found to have a beneficial effect on bone health based on measurements of BMD, *T*-score, and CTx levels in healthy menopausal women, when compared to placebo. These changes were apparent in both the lumbar spine and the femoral neck regions. Combining the CTx data with the BMD analyses strongly indicates that red clover decreases osteoclast activity and promotes osteoblast activity, leading to increase in BMD in the short term. However, no changes in 24-hour blood pressure or markers of inflammation status were found.

## Figures and Tables

**Table 1:** Postfermentation isoflavone composition and quantification analysis of the final extract.

Parameters	Results^a^
Daidzein	7.9 mg/L
Genistein	20.9 mg/L
Biochanin A	45.1 mg
Formononetin	95.0 mg/L
Ononin	9.0 mg
Sissotrin	7.6 mg/L
Dry matter	30.4 g/L

^a^Values are mean.

**Table 2:** The baseline characteristics for treatment group (RC) and placebo.

	RC (*n* = 29)	Placebo (*n* = 31)	*p* value^a^
Age, years	52.65 (±0.85)	52.28 (±0.42)	*p* > 0.05
BMI	26.01 (±0.97)	25.45 (±0.60)	*p* > 0.05
FSH, int.enh./L	72.74 (±4.88)	72.07 (±4.85)	*p* > 0.05
Family history of osteoporosis (yes/no)	6/29	5/26	—
DXA			
BMD lumbar spine, g/cm^2^	1.06 (±0.04)	1.05 (±0.04)	*p* > 0.05
BMD femoral neck, g/cm^2^	0.913 (±0.02)	0.85 (±0.02)	*p* > 0.05
BMC, lumbar spine, g	50.49 (±2.16)	48.34 (±1.57)	*p* > 0.05
BMC, femoral neck, g	4.31 (±0.13)	4.09 (±0.09)	*p* > 0.05
*T*-score, lumbar spine, SD	−0.23 (±0.39)	−0.36 (±0.23)	*p* > 0.05
*T*-score, femoral neck, SD	−0.06 (±0.20)	−0.57 (±0.17)	*p* > 0.05
Biochemical marker			
Ctx ng/mL	0.48 (±0.05)	0.54 (±0.03)	*p* > 0.05
24-hour blood pressure			
(i) Systolic mmHg	125.0 (±1.89)	124.5 (±2.21)	*p* > 0.05
(ii) Diastolic mmHg	78.9 (±1.36)	78.2 (±1.26)	*p* > 0.05

^a^Representing *p* value between groups at baseline. Data are presented as mean (±SEM).

**Table 3:** Systolic and diastolic 24-hour blood pressure before (week 0) and after (week 12) the intervention in the two groups, RC and placebo.

	RC			Placebo		
	Week 0	Week 12	*p* value^a^	Week 0	Week 12	*p* value^a^
24-hour BP: systolic (mmHg)	125.0 (±1.89)	122.6 (±2.17)	*p* > 0.05	124.5 (±2.21)	122.1 (±2.23)	*p* > 0.05
24-hour BP: diastolic (mmHg)	78.9 (±1.36)	77.1 (±1.40)	*p* > 0.05	78.2 (±1.26)	77.3 (±1.43)	*p* > 0.05

^a^Representing *p* value within-group differences between week 0 and week 12. Data are presented as mean (±SEM).

**Table 4:** BMC at lumbar spine and femoral neck before (week 0) and after (week 12) the intervention in the two groups, RC and placebo.

	RC			Placebo		
	Week 0	Week 12	*p* value^a^	Week 0	Week 12	*p* value^a^
BMC: lumbar spine, g	50.49 (±2.16)	50.26 (±2.07)	*p* > 0.05	48.34 (±1.57)	48.89 (±1.77)	*p* > 0.05
BMC: femoral neck, g	4.312 (±0.13)	4.263 (±0.13)	*p* < 0.05	4.087 (±0.10)	4.056 (±0.10)	*p* > 0.05

^a^Representing *p* value within-group differences between week 0 and week 12. Data are presented as mean (±SEM).

**Table 5:** Inflammatory markers before (week 0) and after (week 12) the intervention in the two groups, RC and placebo.

	RC			Placebo		
	Week 0	Week 12	*p* value^a^	Week 0	Week 12	*p* value^a^
IL-1b, pg/mL	16.8 (±0.9)	16.6 (±0.9)	*p* > 0.05	18.3 (±1.2)	15.9 (±1.1)	*p* > 0.05
IL-2, pg/mL	93.9 (±3.7)	93.4 (±4.2)	*p* > 0.05	103.3 (±5.7)	94.9 (±6.6)	*p* > 0.05
IL-4, pg/mL	13.5 (±0.7)	13.3 (±0.7)	*p* > 0.05	15.2 (±0.9)	13.4 (±1.0)	*p* > 0.05
IL-5, pg/mL	101.1 (±6.8)	107.5 (±6.8)	*p* > 0.05	104.4 (±7.9)	97.4 (±8.0)	*p* > 0.05
IL-6, pg/mL	21.5 (±0.9)	22.54 (±1.2)	*p* > 0.05	25.0 (±1.6)	23.2 (±1.6)	*p* > 0.05
IL-10, pg/mL	18.4 (±0.9)	17.4 (±0.8)	*p* > 0.05	19.6 (±1.0)	18.1 (±1.1)	*p* > 0.05
IL-12, pg/mL	18.8 (±0.9)	18.0 (±0.9)	*p* > 0.05	20.5 (±1.1)	18.5 (±1.3)	*p* > 0.05
IL-13, pg/mL	15.5 (±0.9)	16.0 (±0.9)	*p* > 0.05	17.2 (±1.1)	15.5 (±1.1)	*p* > 0.05
IFN-gamma, pg/mL	33.1 (±1.6)	32.3 (±1.6)	*p* > 0.05	35.5 (±1.8)	35.1 (±2.1)	*p* > 0.05

^a^Representing *p* value within-group differences between week 0 and week 12. Data are presented as mean (±SEM).
